# Development and internal validation of a clinical nomogram for identifying lupus nephritis in patients with systemic lupus erythematosus

**DOI:** 10.3389/fmed.2026.1898912

**Published:** 2026-09-16

**Authors:** Xiaojuan Song, Tianshu Chu

**Affiliations:** Department of Rheumatology and Clinical Immunology, Henan Provincial People’s Hospital, Zhengzhou University People’s Hospital, Henan University People’s Hospital, Zhengzhou, Henan, China

**Keywords:** albumin-creatinine index, complement biomarkers, lupus nephritis, nomogram, prediction model, systemic lupus erythematosus

## Abstract

**Objective:**

Lupus nephritis (LN) is one of the most severe manifestations of systemic lupus erythematosus (SLE). This study aimed to develop and internally validate a clinical nomogram based on routinely available clinical and laboratory variables for identifying LN in patients with SLE.

**Methods:**

This retrospective study included 132 patients with SLE after exclusion of one patient with missing LN status from an initial cohort of 133 patients. The C3/C4 ratio was calculated as serum C3 divided by serum C4, and the albumin-creatinine index (ACI) was calculated as serum albumin divided by serum creatinine. The primary multivariable logistic regression model included age, sex, C3/C4 ratio, ACI, and log-transformed anti-dsDNA antibody. Total SLEDAI score was excluded from the primary model because its renal and serological descriptors overlap with the outcome and candidate predictors. The complete-case analysis included 131 patients. Model performance was assessed using receiver operating characteristic analysis, calibration analysis, the Hosmer-Lemeshow test, Brier score, bootstrap internal validation, decision curve analysis (DCA), and clinical impact curve analysis.

**Results:**

Among the 132 eligible patients, 80 (60.6%) had LN and 52 (39.4%) did not. Compared with patients without LN, those with LN had lower C3, C4, albumin, ACI, hemoglobin, and platelet count and higher creatinine, triglyceride, anti-dsDNA antibody, and SLEDAI scores. In the primary multivariable model, a higher ACI was independently associated with lower odds of LN (adjusted OR per 0.1-unit increase = 0.429, 95% CI 0.315–0.586, *p* < 0.001). The C3/C4 ratio (*p* = 0.789) and log-transformed anti-dsDNA antibody (*p* = 0.201) were not independent predictors. The model showed good discrimination (AUC = 0.888, 95% CI 0.830–0.940), acceptable calibration (Hosmer-Lemeshow χ^2^ = 7.451, *p* = 0.489), and a Brier score of 0.132. The bootstrap-corrected AUC was 0.868.

**Conclusion:**

A clinical nomogram based on routinely available variables showed good discrimination after internal validation for identifying LN among patients with SLE. ACI was the only independent predictor in the primary model. The C3/C4 ratio was not independently associated with LN and should be interpreted as an exploratory complement indicator rather than an established predictor. External validation is required before clinical implementation.

## 1 Introduction

Systemic lupus erythematosus (SLE) is a chronic autoimmune disease characterized by multisystem involvement and heterogeneous clinical manifestations. Lupus nephritis (LN) is one of the most severe organ manifestations of SLE and remains an important cause of chronic kidney disease, end-stage renal disease, and mortality (1–4). Early recognition of renal involvement is therefore essential for timely clinical evaluation and treatment.

Kidney biopsy is the reference standard for the diagnosis and pathological classification of LN (5). However, its invasive nature limits its application as a repeated screening tool (3, 6–8). In routine clinical practice, clinicians often rely on conventional serological and biochemical markers to identify patients who require closer renal assessment (9).

The complement system plays a central role in SLE immunopathogenesis. Absolute C3 and C4 concentrations are routinely used to assess complement consumption and disease activity, and recent LN studies have emphasized persistent or isolated hypocomplementemia, particularly low C3, as a prognostic marker (10–13). The C3/C4 ratio may theoretically reflect the relative pattern of complement consumption and has shown prognostic utility in IgA nephropathy (14). However, evidence supporting the ratio in LN is limited, and its potential value should be considered exploratory rather than established. Serum albumin and creatinine are also routinely measured. Hypoalbuminemia may reflect urinary protein loss and systemic inflammation, whereas elevated creatinine may indicate impaired renal function (3, 7). ACI may integrate these two clinically relevant dimensions into a simple serum-derived indicator.

Nomograms translate regression models into individualized risk estimates and have been increasingly used in clinical prediction studies (15–17). This study aimed to develop and internally validate a nomogram based on routinely available variables for identifying LN in patients with SLE (18). The C3/C4 ratio was evaluated as a prespecified exploratory complement indicator, while total SLEDAI score was excluded from the primary model because its renal and serological descriptors overlap with the outcome and candidate predictors (12).

## 2 Materials and methods

### 2.1 Study design and participants

This retrospective study included 133 patients with SLE who were treated at our hospital between October 2023 and September 2025. One patient with missing LN status was excluded, leaving 132 patients for analysis. The inclusion criteria were: (1) fulfillment of the 1997 ACR classification criteria or the 2019 EULAR/ACR classification criteria (19, 20) for SLE; (2) a clearly documented LN status; and (3) availability of the clinical and laboratory variables required for the planned analyses. Patients were eligible irrespective of disease activity status. The exclusion criteria were: (1) coexisting autoimmune disease, primary glomerular disease, active infectious disease, or malignancy; (2) pregnancy or lactation; and (3) use of antibiotics or other anti-infective drugs within one month before enrollment.

### 2.2 Definition of lupus nephritis

The outcome was renal involvement/LN status. LN status was determined according to medical records, clinical assessment, laboratory evidence of renal involvement, and/or renal biopsy results when available (3, 7).

### 2.3 Data collection and derived indicators

Demographic, clinical, treatment, and laboratory variables included age, sex, disease duration, BMI, complement C3, complement C4, blood cell counts, hemoglobin, platelet count, serum albumin, serum creatinine, triglycerides, anti-dsDNA antibody, SLEDAI score, and corticosteroid exposure. The C3/C4 ratio was calculated as serum C3 divided by serum C4; a C4 value of 0 was treated as missing to avoid division by zero. ACI was calculated as serum albumin divided by serum creatinine. Anti-dsDNA values below 1 IU/mL were coded as 0.5 IU/mL and transformed using the natural logarithm of x + 1 [ln(anti-dsDNA+1)].

### 2.4 Statistical analysis

Continuous variables were tested for distributional characteristics. Variables were presented uniformly within each row as mean ± standard deviation or median (interquartile range), according to the overall distribution. Categorical variables were presented as number and percentage. Between-group comparisons were performed using independent-samples t tests, Mann–Whitney U tests, chi-square tests, or Fisher exact tests, as appropriate.

Univariable logistic regression was used to describe crude associations between candidate variables and LN. Candidate predictors for the primary multivariable logistic regression model were selected *a priori* on the basis of clinical relevance and prior evidence rather than by automated stepwise selection. The primary model included age, sex, C3/C4 ratio, ACI, and log-transformed anti-dsDNA antibody. Total SLEDAI score was excluded from the primary model because it contains renal descriptors (proteinuria, hematuria, pyuria, and urinary casts) and serological descriptors (complement and anti-dsDNA antibody) that overlap with the outcome and other predictors (12, 21). Individual C3, C4, albumin, and creatinine values were not entered together with their derived ratios or indices to avoid structural multicollinearity. Multicollinearity among included predictors was assessed using variance inflation factors (VIFs), with VIF > 5 considered indicative of potentially important collinearity. Because the observed ACI range was substantially smaller than one unit, its OR was reported per 0.1-unit increase to provide a clinically interpretable effect estimate. Because the C3/C4 ratio and anti-dsDNA antibody had missing values, complete-case analysis was used and the primary model included 131 patients. A nomogram was constructed from the primary model. Discrimination was assessed using the ROC curve and AUC; calibration was evaluated using a calibration plot, Hosmer-Lemeshow test, and Brier score (22). Internal validation was performed using 1,000 bootstrap resamples. Clinical utility was assessed using decision curve analysis (DCA) and clinical impact curve analysis (23). Statistical tests were two-sided, and *p* < 0.05 was considered statistically significant. Reporting followed recommendations for multivariable prediction model studies (16, 17).

### 2.5 Ethics statement

This human-subject retrospective study was approved by the Ethics Committee of Henan Provincial People’s Hospital (approval no. 2026-012). The requirement for written informed consent was waived because of the retrospective design and use of anonymized data. No animal subjects or animal-derived materials were involved.

## 3 Results

### 3.1 Patient selection and baseline characteristics

The patient selection process is shown in Figure 1. A total of 133 patients were initially screened. After exclusion of 1 patient with missing LN status, 132 patients were included, including 80 patients with LN and 52 without LN.

**Figure 1:**
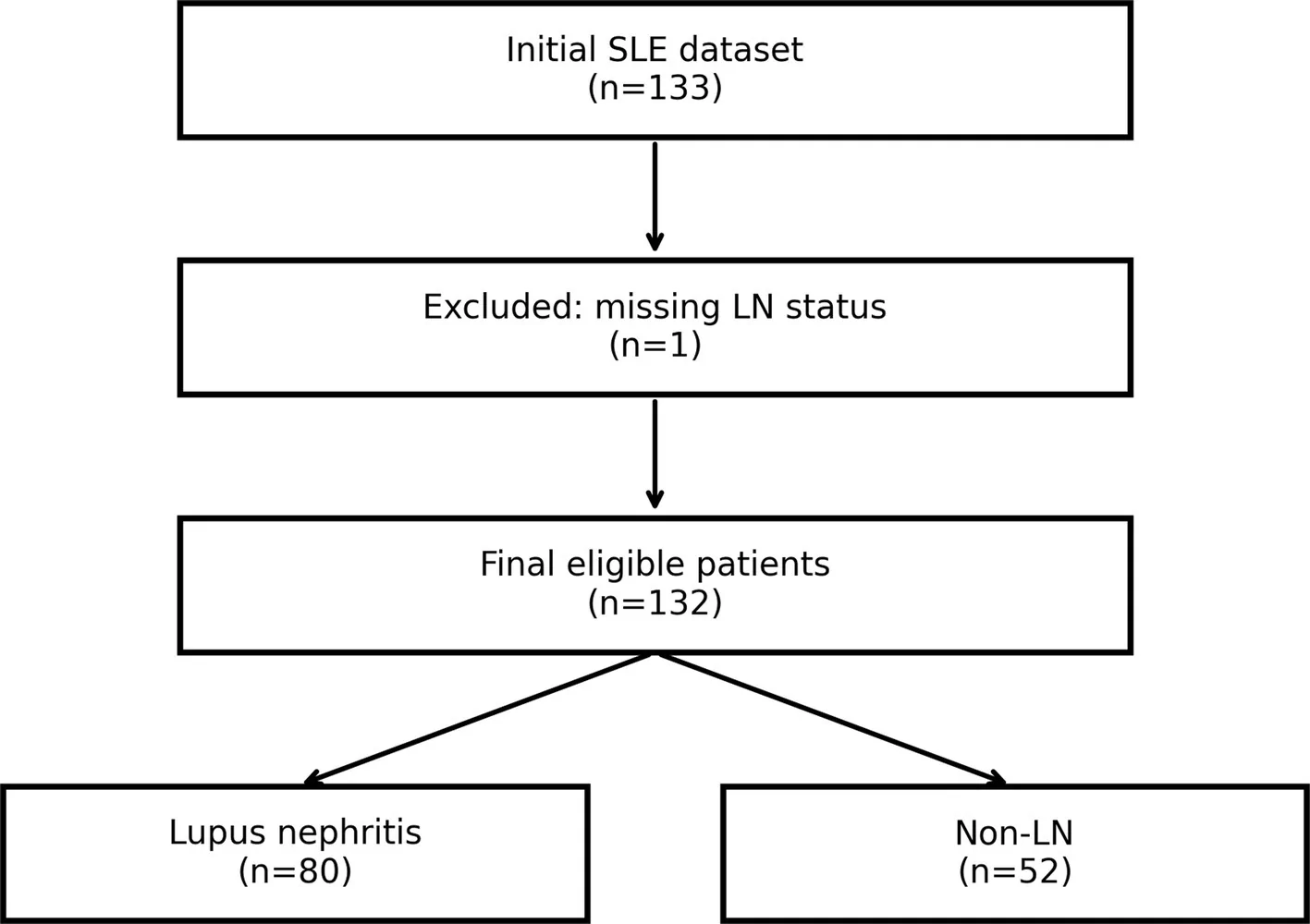
Patient selection flowchart. From 133 initially screened patients, 132 were included in the baseline analysis; 131 complete cases were included in the primary multivariable model.

Baseline characteristics are summarized in Table 1. The distributions of major biomarkers between the LN and non-LN groups are shown in Figure 2. Compared with patients without LN, patients with LN had significantly lower complement C3, complement C4, albumin, ACI, hemoglobin, and platelet count and higher creatinine, triglyceride, anti-dsDNA antibody, and SLEDAI scores (all *p* < 0.05). The C3/C4 ratio did not differ significantly between the groups (*p* = 0.101).

**Table 1:** Baseline characteristics of SLE patients stratified by LN status.

Variable	Total (*n* = 132)	LN (*n* = 80)	Non-LN (*n* = 52)	*p* value
Female, *n* (%)	114 (86.4)	66 (82.5)	48 (92.3)	0.127
Age, years	35.50 (22.00–48.00)	33.00 (21.00–46.25)	39.00 (24.50–51.00)	0.320
Disease duration, months	5.50 (1.00–36.00)	2.00 (1.00–12.00)	12.00 (4.75–36.00)	<0.001
BMI, kg/m^2^	22.75 (20.43–25.65)	23.42 (21.06–25.56)	21.88 (19.88–25.78)	0.276
Complement C3, g/L	0.72 (0.44–0.94)	0.47 (0.39–0.78)	0.89 (0.76–1.04)	<0.001
Complement C4, g/L	0.11 (0.05–0.20)	0.08 (0.03–0.17)	0.17 (0.10–0.22)	<0.001
C3/C4 ratio	6.58 (4.47–9.43)	6.83 (4.58–11.17)	5.84 (4.42–7.77)	0.101
White blood cell count, ×10^9^/L	4.62 (3.41–6.89)	4.42 (3.15–6.09)	4.75 (3.70–7.59)	0.261
Neutrophil count, ×10^9^/L	3.10 (2.22–4.64)	3.08 (2.00–4.16)	3.27 (2.46–4.96)	0.181
Lymphocyte count, ×10^9^/L	1.07 (0.72–1.52)	1.00 (0.71–1.48)	1.14 (0.78–1.67)	0.323
Monocyte count, ×10^9^/L	0.32 (0.21–0.46)	0.33 (0.19–0.46)	0.30 (0.22–0.46)	0.672
Hemoglobin, g/L	107.55 ± 21.64	100.59 ± 22.00	118.27 ± 16.12	<0.001
Platelet count, ×10^9^/L	177.50 (137.00–230.25)	166.00 (124.50–216.00)	206.50 (159.50–246.00)	0.008
Albumin, g/L	34.33 (28.52–38.57)	29.31 (23.00–34.02)	37.73 (35.84–40.28)	<0.001
Creatinine, μmol/L	58.00 (49.83–75.32)	64.20 (54.00–90.65)	53.20 (44.98–57.25)	<0.001
Albumin-creatinine index	0.56 ± 0.26	0.43 ± 0.22	0.77 ± 0.18	<0.001
Triglyceride, mmol/L	1.43 (1.12–2.05)	1.64 (1.25–2.39)	1.22 (0.91–1.56)	<0.001
Anti-dsDNA antibody, IU/mL	73.35 (9.93–292.49)	98.00 (24.86–300.00)	25.10 (3.10–147.38)	<0.001
SLEDAI score	9.00 (4.00–15.25)	13.50 (9.00–16.00)	4.00 (2.00–7.00)	<0.001

Values are presented as mean ± SD, median (IQR), or n (%), as appropriate. LN, lupus nephritis; SLEDAI, Systemic Lupus Erythematosus Disease Activity Index.

**Figure 2:**
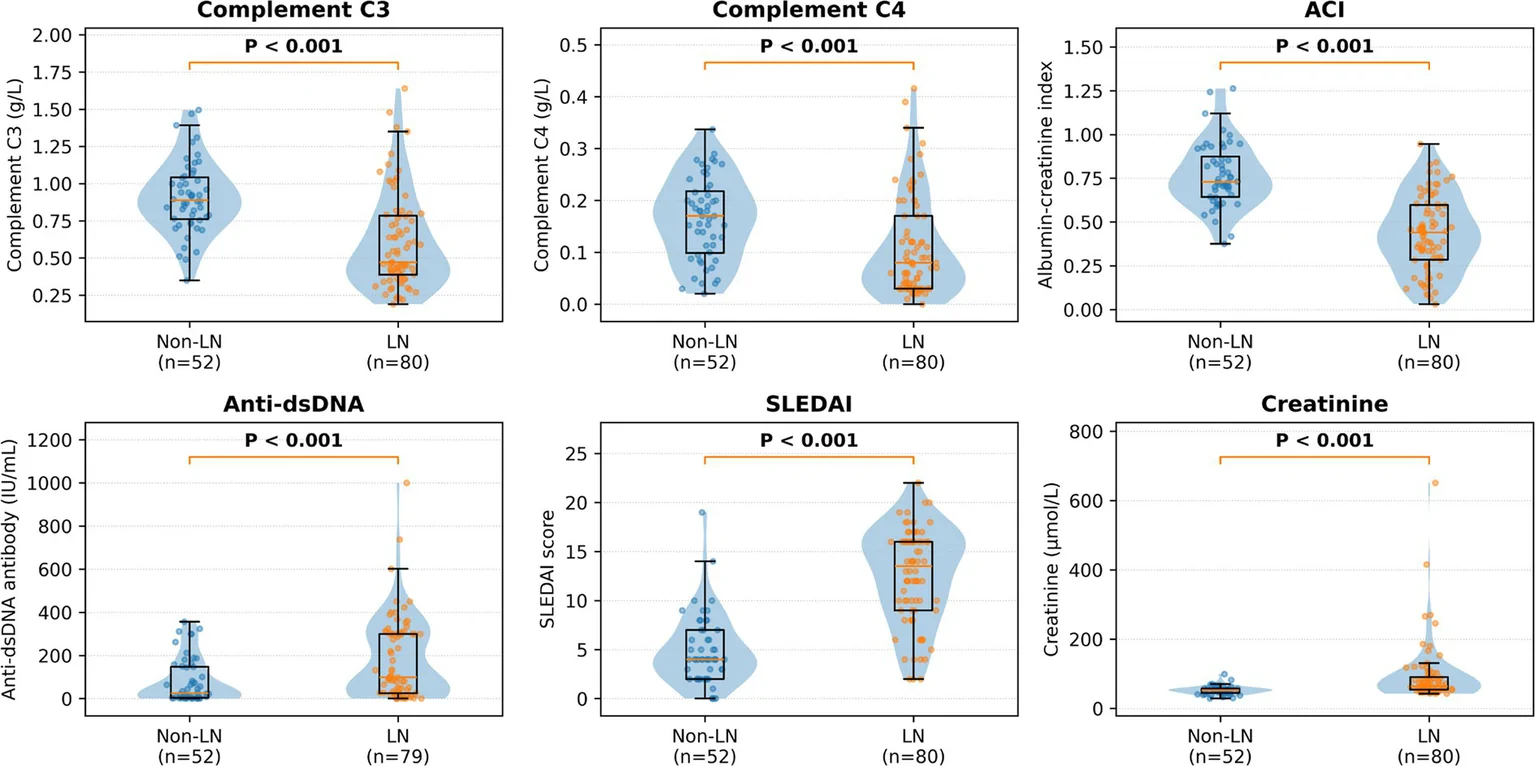
Distribution of major biomarkers between LN and non-LN groups.

### 3.2 Correlation analysis

Spearman correlation analysis is visualized in Figure 3, with detailed coefficients provided. ACI showed a strong negative correlation with LN status (*ρ* = −0.639, *p* < 0.001), whereas SLEDAI score (*ρ* = 0.655, *p* < 0.001), creatinine (ρ = 0.464, *p* < 0.001), triglyceride (ρ = 0.332, *p* < 0.001), and anti-dsDNA antibody (ρ = 0.291, *p* < 0.001) showed positive correlations. The C3/C4 ratio was not significantly correlated with LN status (ρ = 0.144, *p* = 0.100). The association between total SLEDAI score and LN was considered descriptive because the score contains renal and serological descriptors overlapping with the outcome and candidate predictors.

**Figure 3:**
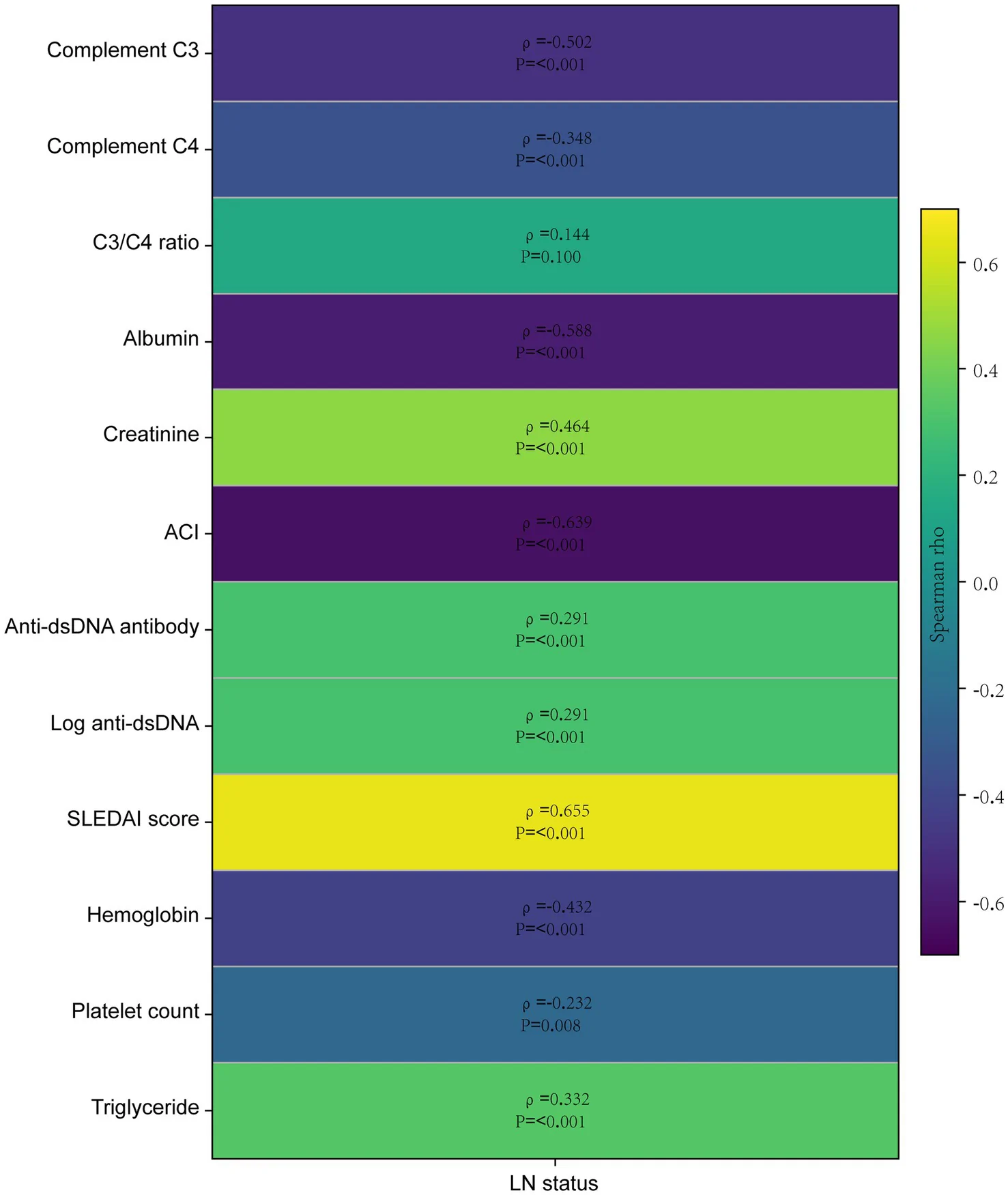
Spearman correlation heatmap among candidate predictors. Data source: Positive rho values indicate higher values in patients with LN; negative rho values indicate lower values in patients with LN (Table S1).

### 3.3 Univariable logistic regression analysis

Univariable logistic regression results are shown in Table 2. Complement C3, complement C4, albumin, creatinine, ACI, anti-dsDNA antibody, SLEDAI score, hemoglobin, platelet count, and triglyceride were associated with LN (all *p* < 0.05). The C3/C4 ratio was not significant (OR = 1.014, 95% CI 0.950–1.082, *p* = 0.674).

**Table 2:** Univariable logistic regression analysis for LN.

Variable	OR	95% CI	*p* value	*n*
Age, years	0.989	0.967–1.011	0.315	132
Female sex	0.393	0.122–1.268	0.118	132
Disease duration, months	0.996	0.989–1.003	0.252	132
BMI, kg/m^2^	1.048	0.956–1.148	0.320	132
Complement C3, g/L	0.032	0.008–0.133	<0.001	132
Complement C4, g/L	0.001	<0.001–0.065	0.001	132
C3/C4 ratio	1.014	0.950–1.082	0.674	131
Albumin, g/L	0.791	0.726–0.862	<0.001	132
Creatinine, μmol/L	1.067	1.034–1.102	<0.001	132
ACI per 0.1-unit increase	0.421	0.311–0.569	<0.001	132
Anti-dsDNA antibody, IU/mL	1.005	1.002–1.008	0.001	131
Log-transformed anti-dsDNA	1.367	1.118–1.671	0.002	131
SLEDAI score	1.441	1.278–1.624	<0.001	132
Hemoglobin, g/L	0.956	0.937–0.976	<0.001	132
Platelet count, ×10^9^/L	0.995	0.991–1.000	0.039	132
WBC, ×10^9^/L	0.960	0.857–1.074	0.474	132
Triglyceride, mmol/L	2.862	1.541–5.315	<0.001	127

ACI is reported per 0.1-unit increase to avoid extremely small OR values and improve interpretability.

### 3.4 Multivariable logistic regression and nomogram construction

The primary multivariable complete-case model included 131 patients and excluded total SLEDAI score. ACI was the only independent predictor: each 0.1-unit increase in ACI was associated with 57.1% lower odds of LN (adjusted OR = 0.429, 95% CI 0.315–0.586, *p* < 0.001; Table 3). The C3/C4 ratio (adjusted OR = 0.989, 95% CI 0.912–1.072, *p* = 0.789) and log-transformed anti-dsDNA antibody (adjusted OR = 1.188, 95% CI 0.913–1.545, *p* = 0.201) were retained as exploratory laboratory indicators but were not independently associated with LN. The corresponding forest plot is shown in Figure 4. A nomogram based on age, sex, C3/C4 ratio, ACI, and log-transformed anti-dsDNA antibody was constructed (Figure 5).

**Table 3:** Multivariable logistic regression analysis for LN.

Variable	B	SE	Adjusted OR	95% CI	*p* value
Age, years	−0.016	0.015	0.984	0.955–1.014	0.292
Female sex	0.065	0.789	1.067	0.227–5.010	0.935
C3/C4 ratio	−0.011	0.041	0.989	0.912–1.072	0.789
ACI per 0.1-unit increase	−0.846	0.158	0.429	0.315–0.586	<0.001
Log-transformed anti-dsDNA(+1)	0.172	0.134	1.188	0.913–1.545	0.201

The primary multivariable model included 131 complete cases and excluded total SLEDAI score to avoid overlap with renal and serological descriptors. ACI is reported per 0.1-unit increase. OR, odds ratio; CI, confidence interval.

**Figure 4:**
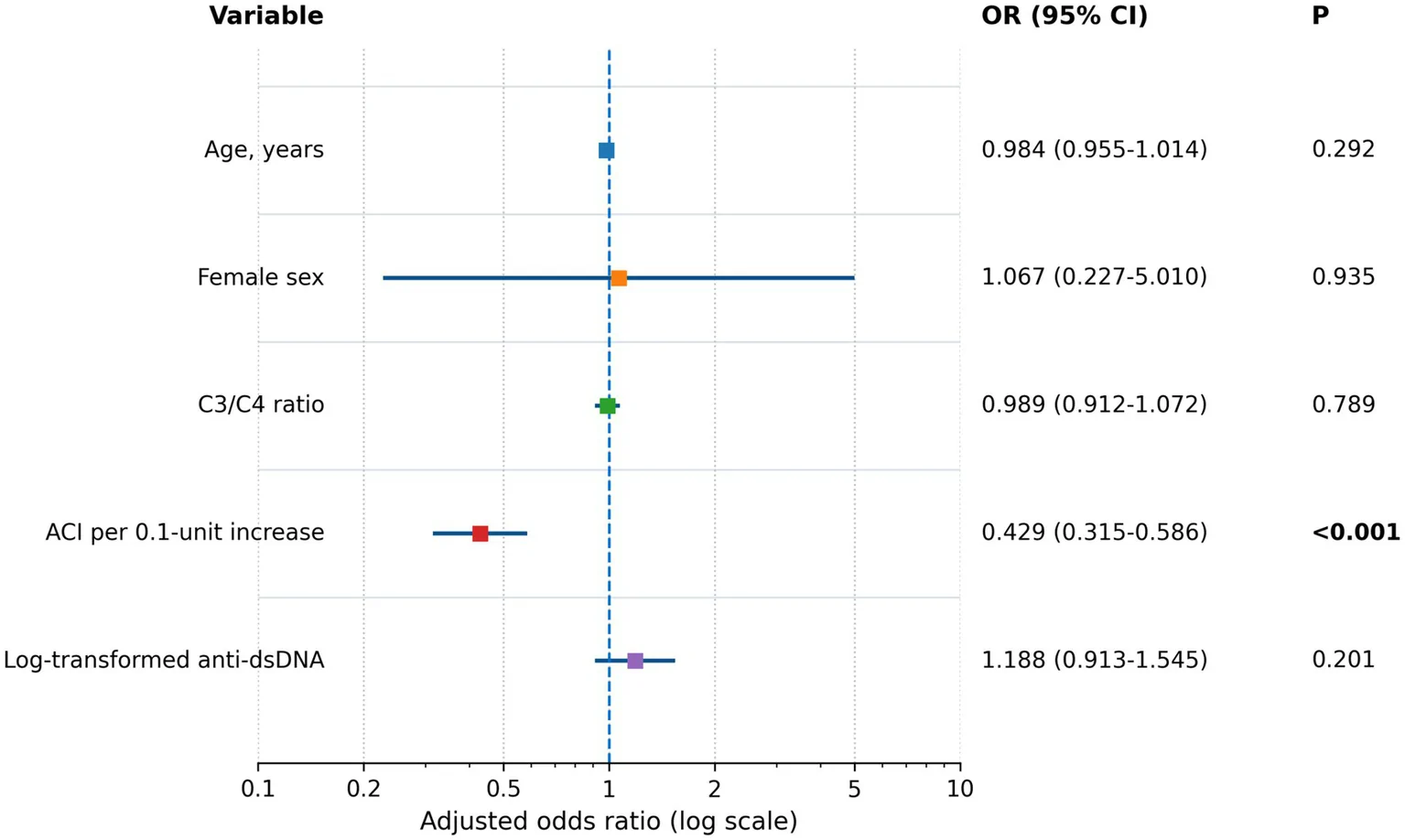
Forest plot of the primary multivariable logistic regression model excluding total SLEDAI score.

**Figure 5:**
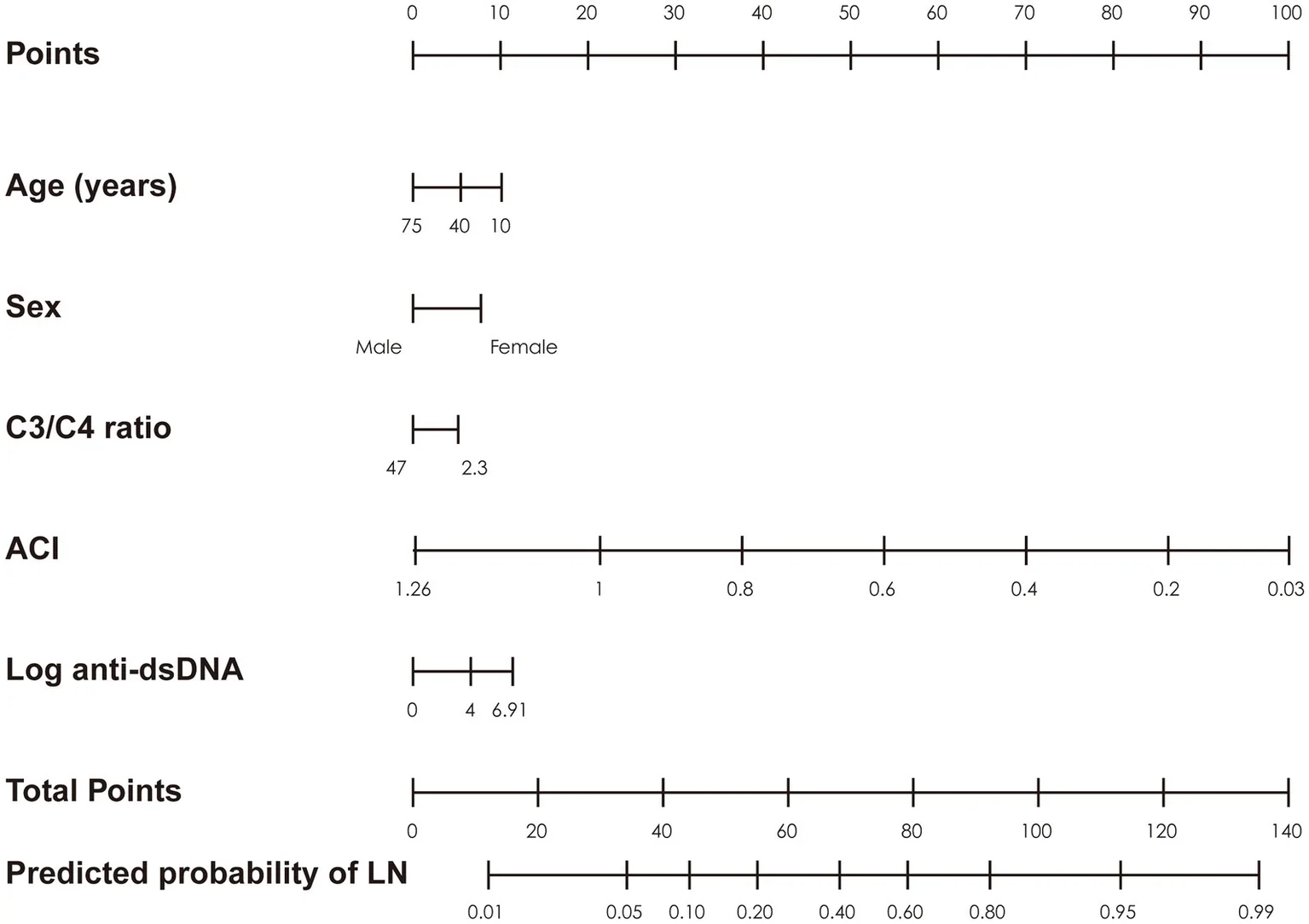
Nomogram for predicting LN based on the primary model excluding total SLEDAI score. ACI, albumin-creatinine index. Axis lengths are scaled to the model-derived maximum point contribution; total SLEDAI score was excluded.

### 3.5 Model performance and internal validation

The primary model showed good discrimination, with an AUC of 0.888 (95% CI 0.830–0.940; Figure 6). Calibration analysis showed acceptable agreement between predicted and observed probabilities (Figure 7). The Hosmer-Lemeshow test showed no significant lack of fit (χ^2^ = 7.451, *p* = 0.489), and the Brier score was 0.132. Internal validation using 1,000 bootstrap resamples produced a corrected AUC of 0.868. Detailed performance indicators are presented in Table 4.

**Figure 6:**
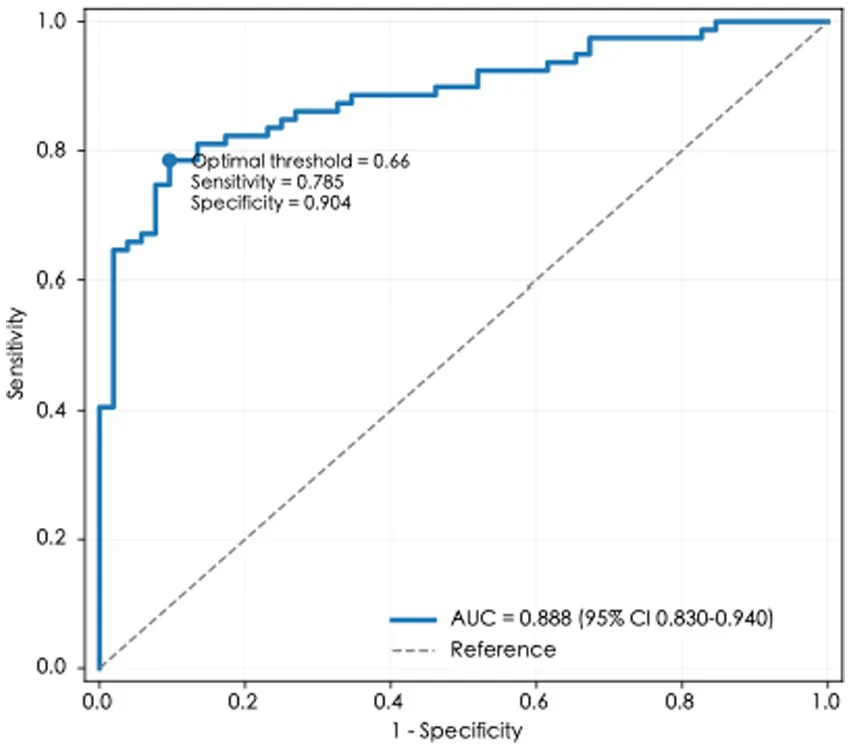
Receiver operating characteristic curve of the primary prediction model.

**Figure 7:**
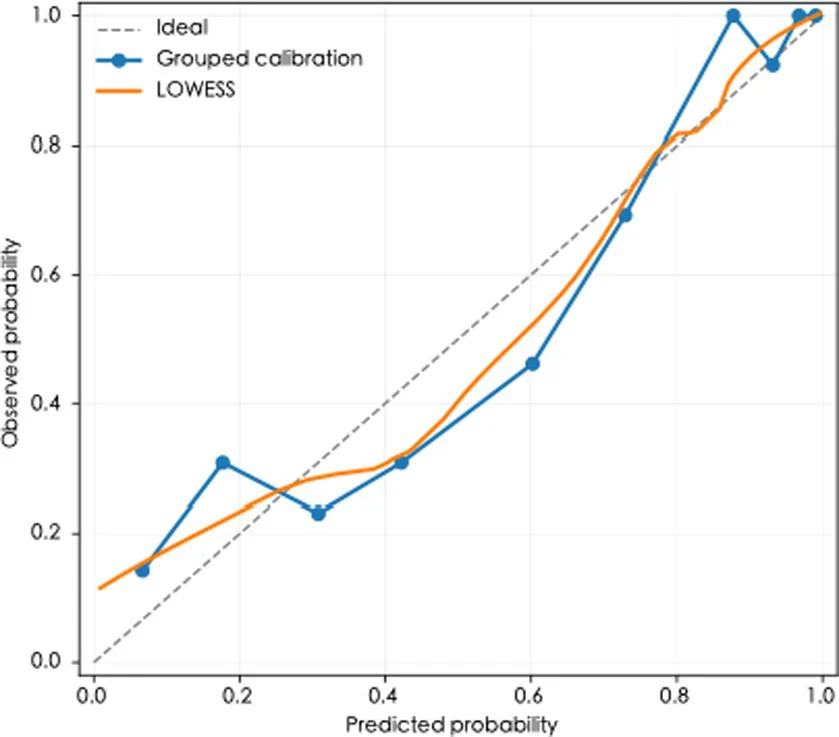
Calibration curve of the primary prediction model.

**Table 4:** Model performance metrics.

Metric	Estimate	Additional information
Complete-case model sample size	131	C3/C4 ratio and log anti-dsDNA complete cases
AUC	0.888	0.830–0.940
Bootstrap-corrected AUC	0.868	992/1000 successful repetitions
Optimal cutoff (Youden index)	0.655	
Sensitivity at cutoff	0.785	
Specificity at cutoff	0.904	
Accuracy at cutoff	0.832	
Positive predictive value	0.925	
Negative predictive value	0.734	
Hosmer-Lemeshow χ^2^	7.451	*p* = 0.489; 10 groups
Brier score	0.132	

Bootstrap-corrected AUC was calculated using 1,000 resamples.

### 3.6 Clinical utility

Decision curve analysis suggested that the primary model provided positive net benefit across a clinically relevant range of threshold probabilities (Figure 8). Clinical impact curve analysis further indicated that the model may help identify patients requiring closer renal evaluation (Figure 9). At the Youden-index cutoff of 0.655, sensitivity was 0.785, specificity was 0.904, and accuracy was 0.832 (Figure 10).

**Figure 8:**
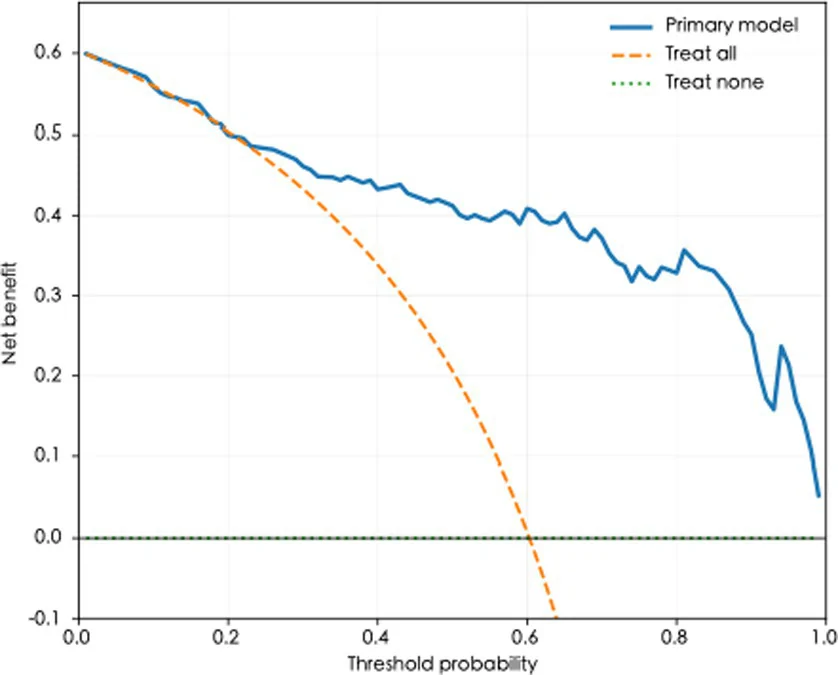
Decision curve analysis of the primary prediction model.

**Figure 9:**
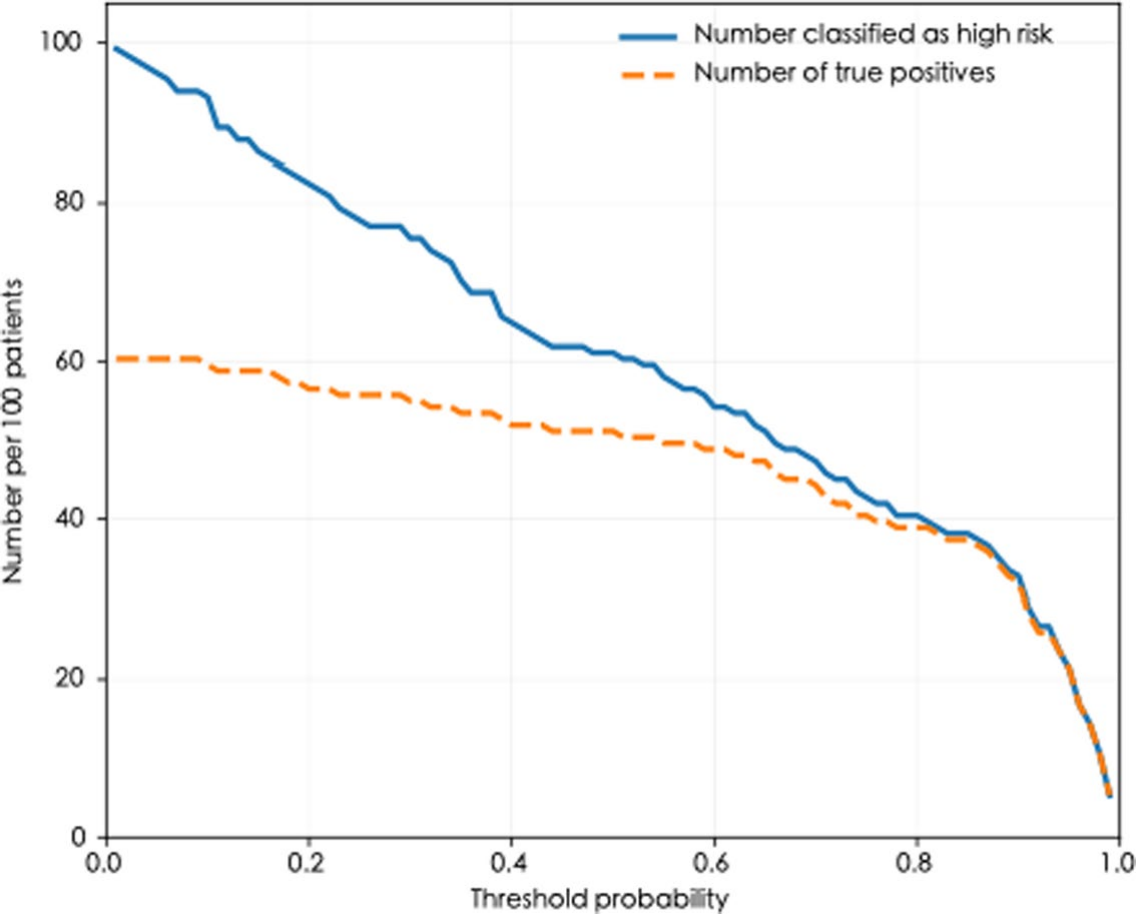
Clinical impact curve of the primary prediction model.

**Figure 10:**
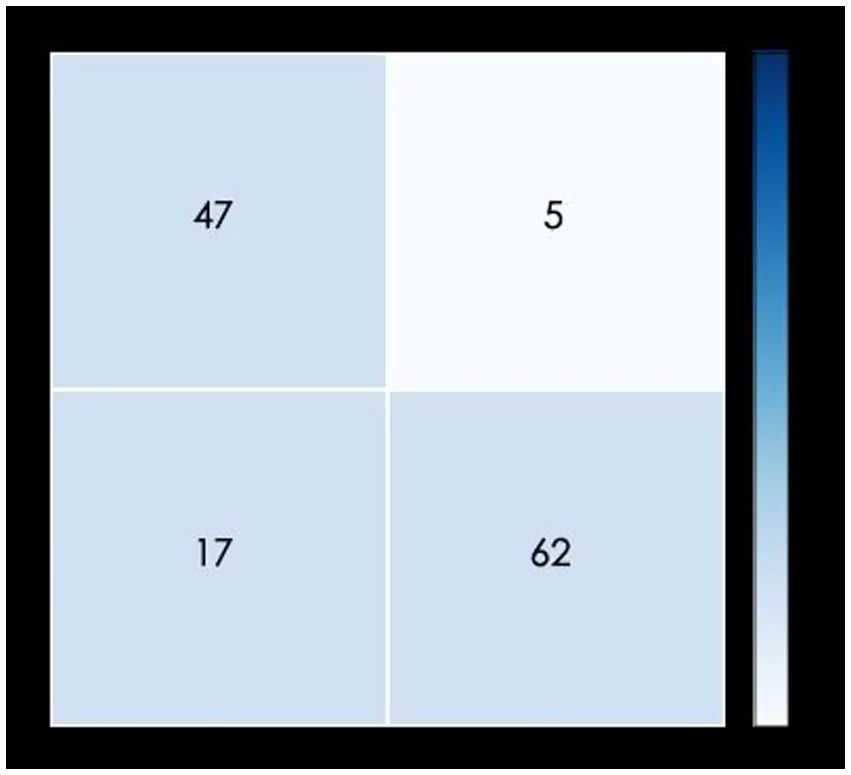
Confusion matrix at the optimal cutoff of the primary prediction model determined by the Youden index.

## 4 Discussion

This study developed and internally validated a nomogram for identifying LN in patients with SLE using routinely available clinical and laboratory variables. The primary model excluded total SLEDAI score to avoid overlap with renal and serological descriptors. ACI was the only independent predictor, whereas the C3/C4 ratio and log-transformed anti-dsDNA antibody were not independently associated with LN. The model showed good discrimination after bootstrap correction, acceptable calibration, and potential clinical utility. These findings should be interpreted as internally validated exploratory results rather than evidence of clinical readiness.

### 4.1 Complement indicators and LN

Complement activation is central to the immunopathogenesis of SLE and LN (11, 13). In the present cohort, absolute C3 and C4 concentrations were significantly lower in patients with LN, supporting the clinical relevance of complement consumption. In contrast, the C3/C4 ratio was not significant in the baseline comparison, correlation analysis, univariable regression, or multivariable regression. Although the ratio may theoretically capture relative patterns of complement consumption and has shown prognostic value in IgA nephropathy (14), recent LN evidence has more commonly supported absolute or persistent hypocomplementemia, particularly isolated low C3, rather than the C3/C4 ratio (24). In this study, the ratio was retained because of exploratory clinical interest, not because it demonstrated statistical significance. Its inclusion in the nomogram should therefore not be interpreted as evidence of independent or incremental predictive value. Future external studies should compare models with and without the ratio and formally assess its incremental contribution.

### 4.2 Clinical meaning of ACI

ACI was the only independent predictor of LN in the primary model. Because ACI combines serum albumin and creatinine, it may reflect both urinary protein loss or systemic inflammation and renal functional impairment (3, 7, 25). Reporting the OR per 0.1-unit increase provides an effect estimate that is more interpretable within the observed clinical range than an OR per one-unit increase. However, because albumin and creatinine may already reflect established renal involvement, ACI should be viewed as a marker for identifying concurrent LN rather than as evidence of future causal prediction (26).

### 4.3 Disease activity and model performance

Total SLEDAI score was higher in patients with LN and correlated with LN status in descriptive analyses. However, total SLEDAI was not included in the primary model because it contains renal descriptors such as proteinuria, hematuria, pyuria, and urinary casts, as well as serological descriptors related to complement and anti-dsDNA antibody (12). Including total SLEDAI together with the outcome-related and serological variables could introduce incorporation bias and artificially inflate model performance. Therefore, the primary model focused on routinely available variables without total SLEDAI. A model including total SLEDAI, if presented, should be clearly labeled as a sensitivity analysis rather than the primary model.

### 4.4 Clinical implications

The proposed nomogram may support renal risk stratification, outpatient triage, and decisions regarding closer renal evaluation. All variables are routinely available, which may facilitate use in clinical settings. Nevertheless, this model identifies the presence of LN in a retrospective cross-sectional clinical context; it does not predict incident LN over time. The nomogram should not replace renal biopsy, longitudinal clinical assessment, or specialist judgment.

### 4.5 Limitations

This study has several limitations. First, it was a single-center retrospective study with a modest sample size (27). Second, the primary multivariable model was based on 131 complete cases rather than the full cohort because of missing or noncomputable C3/C4 ratio and anti-dsDNA values. Third, renal biopsy confirmation was not available for all patients, which may have introduced outcome misclassification. Fourth, the C3/C4 ratio was retained for exploratory clinical interest but was not statistically significant and its incremental value was not established. Fifth, total SLEDAI was excluded from the primary model because item-level nonrenal clinical SLEDAI could not be reconstructed from the available dataset. Sixth, corticosteroid dose or cumulative exposure was not consistently available and therefore could not be included as a covariate. Seventh, the model was developed to identify concurrent LN and cannot establish temporal or causal prediction. Finally, external validation in larger, multicenter cohorts is required before clinical implementation (16, 17).

## 5 Conclusion

A clinical nomogram based on routinely available variables showed good discrimination after internal validation for identifying LN among patients with SLE. ACI was the only independent predictor in the primary model. The present data did not support the C3/C4 ratio as an independent predictor; it should be regarded as an exploratory complement indicator. External validation and assessment of incremental value are required before clinical implementation.

## Data Availability

The datasets presented in this study can be found in online repositories. The names of the repository/repositories and accession number(s) can be found in the article/supplementary material.
